# Cecal cyst as a lead point for intussusception in children: a case report

**DOI:** 10.3389/fped.2026.1904282

**Published:** 2026-09-21

**Authors:** Batool Sawan, Leen Saleh, Dalia Abdulrahman, Mira Alredha, Ghadir Jaber, Diary Mohammad

**Affiliations:** 1 Mohammed Bin Rashid University of Medicine and Health Sciences, Dubai, United Arab Emirates; 2 College of Medicine, Dubai Medical University, Dubai, United Arab Emirates; 3 Pediatric Surgery Department, Al Jalila Children's Specialty Hospital, Dubai, United Arab Emirates

**Keywords:** cecal duplication, infant, intussusception, pneumatic reduction, surgical excision

## Abstract

Enteric duplication cysts are rare congenital malformations that can occur anywhere along the gastrointestinal tract and, although usually asymptomatic, may occasionally act as a pathological lead point for intussusception. We report a case of a cecal duplication cyst presenting as the lead point for ileocecal intussusception in a previously healthy 4-month-old female infant, who presented with acute-onset recurrent vomiting. Abdominal examination revealed a firm, palpable 5-cm mass in the epigastric region, and imaging confirmed intussusception at the ileocecal region with an associated cystic lesion. Following three failed attempts at pneumatic reduction, the patient underwent emergency laparoscopic-assisted reduction of the intussusception with excision of the cecal duplication cyst. Her postoperative course was uneventful, and she was discharged home in good condition. This case underscores the importance of prompt recognition and timely surgical management of duplication cysts presenting as intussusception, given the rarity of this condition and the critical role of early intervention in achieving a favorable outcome.

## Introduction

Duplication cysts are rare congenital malformations that can occur anywhere along the gastrointestinal tract, with cecal duplications accounting for only 0.4% of all reported cases. They typically present in early childhood, although their etiology remains uncertain. Clinical features vary according to the cyst's location, size, and associated complications, and may include abdominal pain, vomiting, intestinal obstruction, intussusception, or gastrointestinal bleeding (1, 2).

While most cases of pediatric intussusception are idiopathic, a pathological lead point is identified in approximately 2%–12% of cases. Meckel's diverticulum is the most common, followed by polyps, enlarged mesenteric lymph nodes, and tumors such as non-Hodgkin lymphoma. Enteric duplication cysts are a rare but increasingly recognized cause and can act as lead points when they arise within or adjacent to the bowel wall, particularly at the ileocecal region (2).

Duplication cysts capable of acting as a lead point are typically small, mucosa-lined cystic or tubular structures that share a common wall and blood supply with the adjacent bowel, allowing them to be drawn into the intussusceptum during peristalsis. Because their clinical presentation is nonspecific and often indistinguishable from that of idiopathic intussusception, duplication-cyst-associated intussusception is easily overlooked and is frequently diagnosed only intraoperatively or on histopathology, particularly when non-operative reduction is unsuccessful or incomplete. Ultrasonography remains the key diagnostic tool in this setting, as identification of a cystic lesion within or adjacent to the intussusceptum should raise suspicion for a duplication cyst as the underlying lead point rather than a purely idiopathic process.

Herein, we report a case of a cecal duplication cyst acting as the lead point for ileocecal intussusception in a 4-month-old female infant, who required surgical intervention following failed pneumatic reduction. To the best of our knowledge, this represents the first such case reported from the United Arab Emirates. We describe the clinical presentation, diagnostic workup, and management of this case, alongside a narrative review of previously published cases, with the aim of increasing awareness of this rare but clinically important cause of pediatric intussusception.

## Case presentation

A previously healthy 4-month-old female infant presented to the emergency department with acute-onset recurrent vomiting and a history of fever three days prior to presentation, which had since resolved. The vomiting began the evening before presentation, occurred approximately six times, consisted of milk content, and was non-projectile and non-bloody. Bowel habits were normal, with the last bowel motion occurring the morning before presentation and no blood or mucus noted. There were no other associated symptoms.

The infant, who was exclusively breastfed, was born at term via spontaneous vaginal delivery at a private hospital, with an uncomplicated antenatal and postnatal course and a normal antenatal ultrasound. There was no significant past medical history, recent vaccination, or relevant family history.

On examination, she was vitally stable and not in acute distress, with no signs of dehydration. In the emergency department, the child's mother attempted to feed her prior to medical assessment; however, the infant was unable to tolerate oral intake. Abdominal examination revealed a firm, palpable mass in the epigastric region, which was non-tender with no overlying erythema or skin discoloration. The remainder of the examination was unremarkable. Following initial evaluation, the patient was kept nil per os (NPO) in anticipation of further diagnostic workup and possible surgical intervention.

Initial laboratory investigations, including complete blood count, C-reactive protein, procalcitonin, urea and electrolytes, and urinalysis, were within normal limits. An abdominal x-ray was performed, which demonstrated a mid-abdominal soft tissue mass with a meniscus sign (Figure 1), suggestive of intussusception, with no evidence of pneumoperitoneum or bowel dilatation.

**Figure 1:**
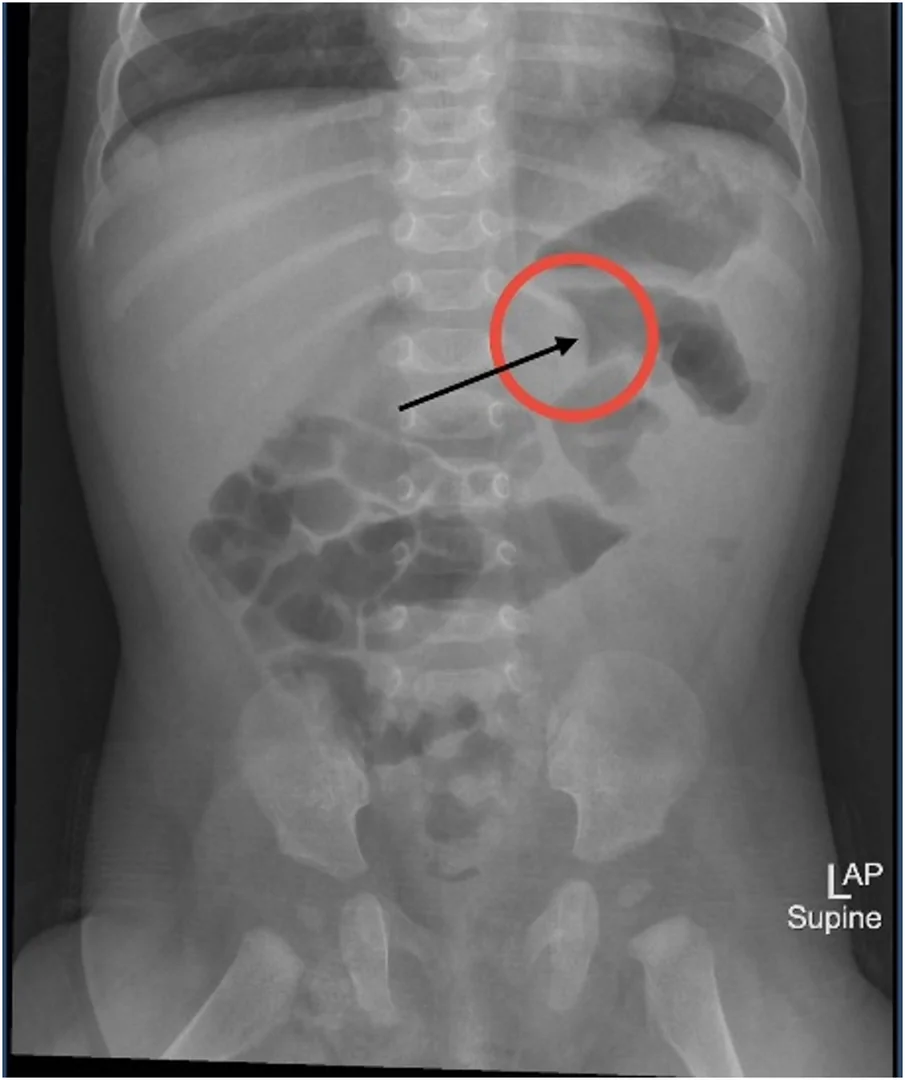
Abdominal x-ray demonstrating an oval opacity projecting at the level of T12–L1 with a meniscus sign, suggestive of intussusception.

Departmental ultrasound imaging confirmed the diagnosis and revealed a central, round, anechoic cystic lesion within the intussuscepted bowel segment, measuring 1.43 × 2.30 cm, with posterior acoustic enhancement, surrounded by a thin layer of mesentery and bowel (Figure 2A). A target sign was identified at the ileocecal region, measuring 6.1 × 2.8 × 2.3 cm (Figure 2B), and a transverse section demonstrated the classic “pseudo-kidney” sign of intussusception (Figure 2C). Color Doppler demonstrated preserved vascular flow within the intussusception complex, arguing against bowel ischemia at this stage.

**Figure 2:**
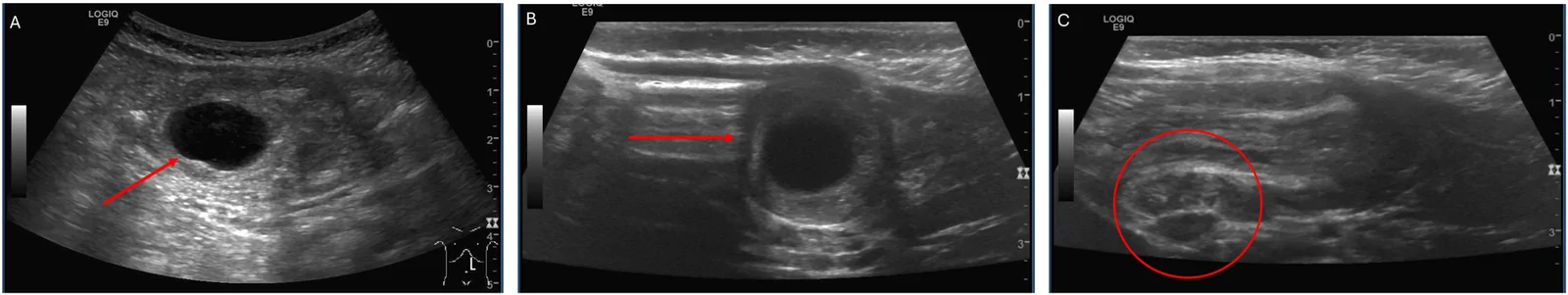
**(A)** The intussusception demonstrates a round, anechoic cystic lesion centrally. **(B)** Target sign representing intussusception identified at ileocecal region. **(C)** Transverse section showing “pseudo kidney” sign of intussusception.

Based on these findings, the patient was transferred for fluoroscopy-guided pneumatic reduction. A Foley catheter was placed and secured in the rectum by the surgical team, and air was insufflated under pressure control through the catheter over three reduction attempts. Despite these efforts, a persistent filling defect remained (Figure 3), with no air seen entering the terminal ileum or small bowel loops. The procedure was deemed unsuccessful, and the rectal tube was removed.

**Figure 3:**
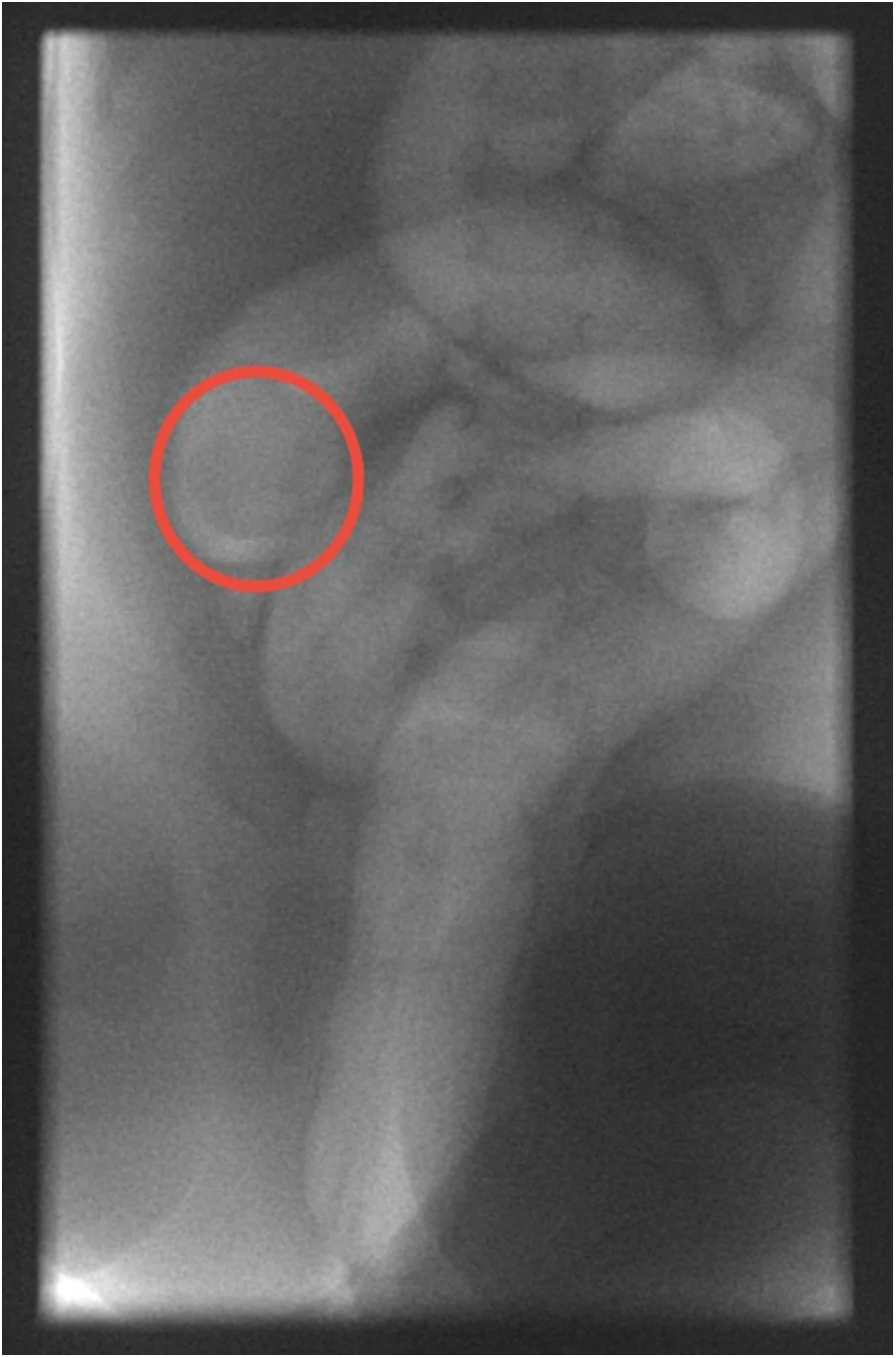
Hydrostatic enema reduction of intussusception, showing reduction till the cecum and a rounded filling defect identified in the cecum.

Following the incomplete reduction, the patient was admitted to the surgical ward for planned surgical intervention. Under general anesthesia, with the patient supine, the abdomen was cleaned and draped; no mass was palpable externally at this stage. A 5-mm laparoscopic camera was inserted through an infraumbilical incision. Laparoscopy revealed a reducible intussusception, with engorgement and dusky discoloration of the appendix and ascending colon, and a mass identified within the cecum. The terminal ileum and cecum were exteriorized through the infraumbilical incision, and the cecum was opened, revealing a cystic lesion on its posterior wall (Figure 4A). The cyst was incised and its wall excised and sent for histopathological examination; the cyst cavity edges were then sutured, the remaining cystic lining cauterized, and the cecum closed using 5-0 Novosyn suture material (Figures 4B–D). Given the dusky appearance of the appendix and signs suggestive of early appendicitis, an appendectomy was also performed.

**Figure 4:**
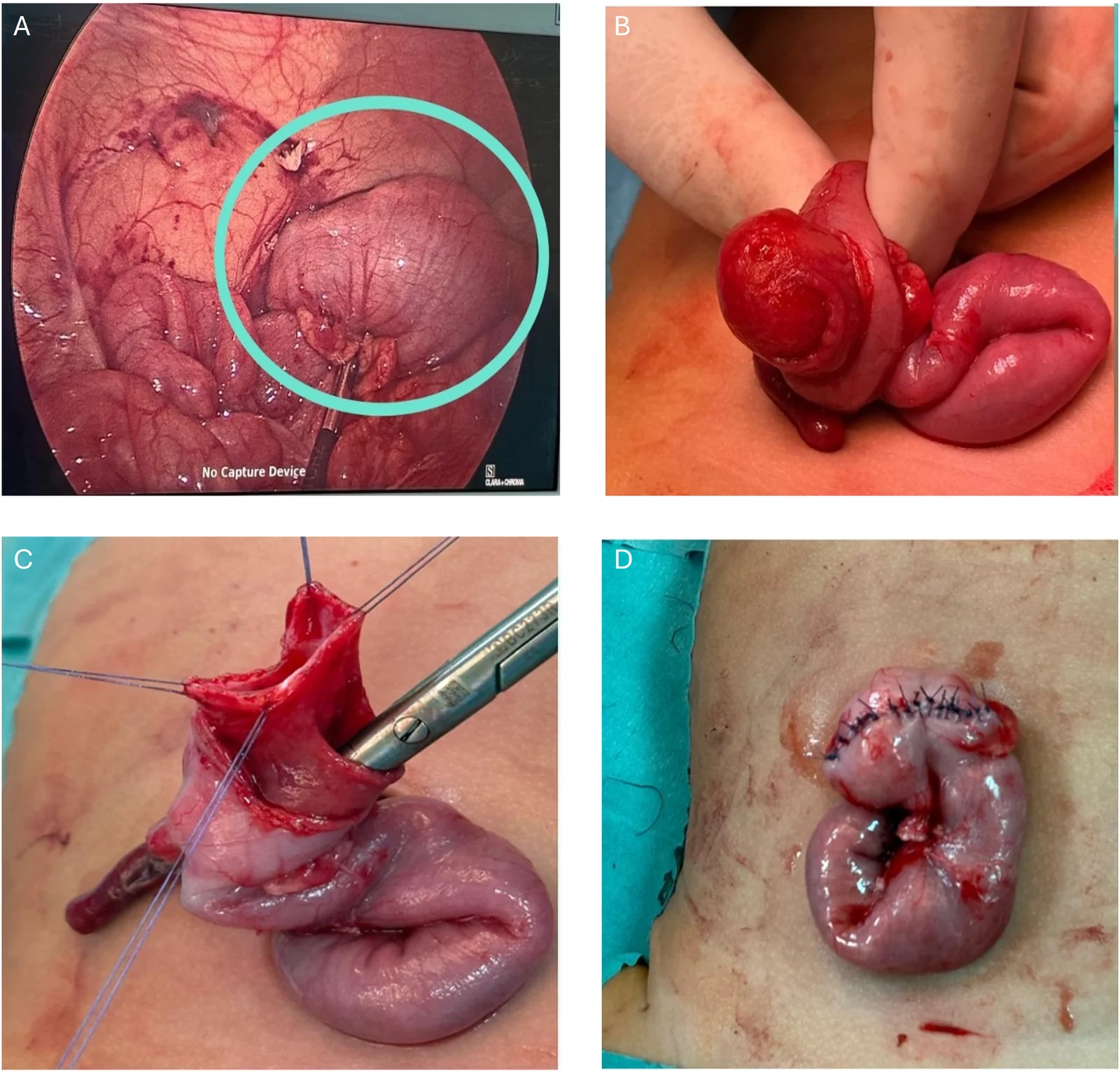
**(A)** Laparoscopic view of the cecal duplication cyst following laparoscopic reduction of the intussusception. **(B)** Intraoperative finding of the cecal duplication cyst after exteriorization of the cecum. **(C)** Marsupialization of the cecal duplication cyst. **(D)** Closure of the cecal enterotomy.

Postoperatively, the patient remained hemodynamically stable. A nasogastric tube was inserted with scheduled three-hourly aspiration, and intravenous Tazocin was commenced every eight hours. She tolerated the procedure well and was discharged safely after six days.

Final histopathological examination demonstrated peri-appendicitis without significant mucosal or mural inflammation. The excised cecal lesion showed colonic and fibroconnective tissue with mixed inflammatory infiltrates, congestion, mild fibrosis, and reactive changes; no intact or well-formed cystic structure was identified in the examined sections, and there was no evidence of dysplasia or malignancy. These findings were interpreted as reactive inflammatory changes, with the absence of a well-defined cyst on histology likely reflecting the marsupialized and partially excised nature of the lesion.

## Discussion

Duplication cysts are rare congenital malformations that can occur throughout the gastrointestinal tract. Colonic duplications represent approximately 3% to 20% of cases (2), with ileal involvement observed in about one-third. Cecal duplications are exceedingly rare, accounting for only 0.4% of all alimentary tract duplications (2) and are more common in females (3).

The exact etiopathogenesis of these malformations remains unclear (4). Several theories have been proposed, including notochordal splitting during embryonic development, persistence of fetal enteric diverticula, intrauterine vascular events, and incomplete recanalization of the intestinal lumen (3). Enteric duplications can present in cystic, tubular, or mixed forms. While cystic duplications of the colon are typically isolated anomalies, tubular duplications of the colorectal region are more frequently associated with genitourinary, vertebral, and other congenital malformations (5). Histologically, duplication cysts display a smooth muscle layer continuous with the adjacent intestinal wall and share its blood supply, along with an inner mucosal lining that often mirrors native bowel mucosa or, less commonly, contains heterotopic elements (5). In addition, ectopic gastrointestinal mucosa – most commonly gastric – is present in about 30% of cases and can result in complications such as gastrointestinal bleeding or perforation. In such cases, technetium-99 m pertechnetate scintigraphy may aid diagnosis by detecting ectopic gastric tissue (2). While typically benign, malignant transformation has occasionally been reported, including adenocarcinoma arising in ileal and colonic duplication cysts (6, 7), underscoring the importance of close histopathological evaluation to ensure oncologic safety.

The clinical manifestations of duplication cysts are influenced primarily by their size and location (8). Smaller cysts often act as pathological lead points, precipitating conditions such as intussusception or volvulus, while larger cysts tend to exert a mass effect that can result in bowel obstruction or, in severe cases, ischemia (8, 9). Most enteric duplication cysts are asymptomatic and are found incidentally (10); when symptomatic, patients with cecal duplication may present with recurrent abdominal pain, vomiting, gastrointestinal bleeding, or constipation.

Ultrasonography is the preferred initial imaging modality for suspected duplication cysts, particularly in the pediatric age group, as it is noninvasive, widely available, and highly accurate (11). A hallmark feature is the “double-wall” or “gut signature” sign, which appears as a hyperechoic inner layer representing the mucosa–submucosa complex, surrounded by a hypoechoic outer rim corresponding to the muscular layer (12). This layered appearance closely mimics the structure of normal bowel wall and is considered highly suggestive of an enteric duplication cyst. In some cases, additional findings, such as a multilayered cyst wall or a Y-shaped configuration at the junction with adjacent bowel, further support the diagnosis. While cross-sectional imaging with computed tomography (CT) or magnetic resonance imaging (MRI) is not routinely required, these modalities may be employed when ultrasound findings are equivocal, when the cyst lies in an anatomically challenging region, or when detailed delineation of its relationship to adjacent structures is needed for surgical planning (11).

Intussusception is generally treated with pneumatic or hydrostatic enema under ultrasound or fluoroscopic guidance, with a reported success rate of 85%–90% (10). When enema reduction is unsuccessful after repeated attempts, surgical intervention becomes necessary. In the case of a cecal duplication cyst, complete surgical excision is the preferred treatment; when full removal is not feasible, segmental resection of the affected bowel with primary anastomosis may be required (10). Management in our case can be considered in two aspects: treatment of the intussusception and management of the underlying duplication cyst. Following three failed pneumatic reductions, surgical resection and bowel repair were performed, consistent with recommendations for definitive management (13).

A narrative review of the literature identifies fewer than a dozen previously reported cases in which an enteric duplication cyst has been implicated as the lead point for intussusception, most occurring in infants and young children. Lai et al. described a boy in whom diagnosis was delayed for three months, having initially been attributed to constipation; at surgery, the duplication cyst was found to have been drawn retrograde through the ileocecal valve, with only mild bowel ischemia despite the prolonged course (1). A similar diagnostic overlap was reported by De Biagi et al., who documented cecocolic intussusception in a young infant with a cecal duplication cyst, in whom intraoperative diagnosis followed by definitive surgical management resulted in a favorable outcome (2). Khalid et al. presented a comparable case of a cecal duplication cyst mimicking intussusception in a female patient, further reinforcing this diagnostic overlap (3). In contrast, Radhakrishna et al. described a five-year-old boy with a week-long history of abdominal pain and ileocolic intussusception that could only be partially reduced hydrostatically; laparotomy in this case revealed a submucosal cecal mass with enlarged mesenteric lymph nodes, which was resected with clear margins (7). A more unusual course was documented by Ghamari Khameneh et al., whose 18-month-old patient developed a cecal-duplication-cyst-associated intussusception that prolapsed transanally — a rare presentation that contributed to diagnostic delay (8). Yammouri et al., meanwhile, reported a four-year-old girl with intermittent abdominal pain and right-lower-quadrant tenderness but no vomiting, whose imaging findings were initially thought to represent appendicitis before an ileocolic intussusception caused by a cecal duplication cyst was identified (9). A case with an almost identical clinical picture was described by Alomar et al., involving a three-year-old child with an enteric duplication cyst of the cecum presenting as ileocolic intussusception (10). Presentations in infants tend to be even less specific: Alfayez and Skef reported a 41-day-old boy with progressive bilious vomiting and abdominal distension, in whom imaging raised suspicion for ileocolic intussusception and small bowel obstruction, with a cecal duplication cyst identified only at surgical exploration and managed by extramucosal enucleation (14). Finally, Kaushal et al. described an 11-month-old infant initially misdiagnosed with gastroenteritis, whose duplication cyst was missed on imaging owing to a malpositioned cecum and was confirmed only intraoperatively, illustrating how anatomical variation can further complicate preoperative diagnosis (11). Collectively, these reports highlight a consistent pattern: duplication-cyst-associated intussusception is difficult to distinguish preoperatively from idiopathic disease, is frequently identified only after failed non-operative reduction or at surgery, and nonetheless carries an excellent prognosis when managed with timely excision.

Although cecal duplication cysts are exceptionally rare, approximately 45 cases have been reported in the literature, with only about 10 presenting as, or being initially mistaken for, intussusception (2, 3, 7). This highlights the diagnostic challenge and the importance of considering a pathological lead point in cases of failed non-operative reduction. Early detection of duplication cysts helps prevent major complications, and surgical resection remains the treatment of choice, with most cases demonstrating excellent postoperative outcomes (14). To the best of our knowledge, there are no previously published reports from the United Arab Emirates describing intussusception secondary to a cecal duplication cyst in an infant, and published reports overall highlight the extreme rarity of this presentation, with only isolated neonatal and infant cases documented globally. This case expands the limited regional data, underscores the need for heightened diagnostic awareness of rare pathological lead points in pediatric intussusception, and reinforces the critical role of timely surgical intervention in achieving optimal outcomes.

## Conclusion

This case illustrates that a cecal duplication cyst, although rare, is a clinically important pathological lead point for intussusception in infants – an entity that remains underrecognized because of its nonspecific presentation and its overlap with more common, idiopathic causes of pediatric intussusception. Our experience reinforces that persistent or recurrent intussusception, particularly following failed non-operative reduction, should raise suspicion for an underlying structural lead point such as a duplication cyst, which is best identified on ultrasonography through features such as a cystic lesion within the intussusceptum or the classic “gut signature” appearance. Prompt surgical exploration and complete excision remain the cornerstone of management and are associated with excellent outcomes when performed in a timely manner. By reporting this case alongside a review of the existing literature, we aim to contribute to the limited body of evidence on duplication-cyst-associated intussusception and to encourage clinicians to maintain a high index of suspicion for this rare but treatable condition in infants presenting with atypical or refractory intussusception.

## Data Availability

The datasets presented in this article are not readily available because access to the data has to be through the Institutional Review Board or the corresponding author. Requests to access the datasets should be directed to LS, leensaleh1@gmail.com.
